# A translation and preliminary validation of the Dutch Wound-QoL questionnaire

**DOI:** 10.1186/s12895-020-00101-2

**Published:** 2020-08-26

**Authors:** Stella F. Amesz, Toni M. Klein, Audrey M. Meulendijks, Tuong-Vi Nguyen, Christine Blome, Petrie F. Roodbol, Catherine van Montfrans

**Affiliations:** 1 University of Groningen, Faculty of Medical Sciences, Kerkstraat 4, 8162RS Epe, the Netherlands; 2 German Centre for Health Services Research in Dermatology (CVderm), Institute for Health Services Research in Dermatology and Nursing (IVDP), University Medical Center Hamburg-Eppendorf, Hamburg, Germany; 3 Research Group Healthy and Sustainable Living, HU University of Applied Sciences, Utrecht, the Netherlands; 4 Department of Dermatology, Erasmus University Medical Center, Rotterdam, The Netherlands; 5 University of Groningen, University Medical Center Groningen, Groningen, The Netherlands

**Keywords:** Chronic wounds, Validation, Dutch Wound-QoL questionnaire, Wound-QoL, EQ-5D-3L

## Abstract

**Background:**

Chronic wounds have a major impact on patients’ health-related quality of life (HRQoL). Therefore, measuring HRQoL is an indispensable part of the treatment of patients with chronic wounds. The aim of this study was to translate and validate the Wound-QoL, a wound-specific HRQoL questionnaire, in a Dutch population.

**Methods:**

The Wound-QoL was translated into Dutch according to the international standards. Patients with chronic wounds were asked to complete questionnaires at baseline (T0) and after six weeks (T1), including Wound-QoL, EQ-5D-3L (a generic questionnaire to measure HRQoL) and a visual analogue scale (VAS) measuring wound pain. If patients were not able to complete the questionnaire by themselves, it was read out to them by a nurse. Further data were obtained from medical records.

**Results:**

Of the 120 patients included, 64 (53.3%) completed the questionnaire by themselves. To 55 patients (45.8%), the questionnaire was read out. The internal consistency of the Wound-QoL global score was high at both time points (T0: Cronbach’s α = 0.89, T1: Cronbach’s α = 0.92). The item selectivity for global score ranged from r = 0.25 to r = 0.77 at T0 and from r = 0.40 to r = 0.79 at T1. Overall, the self-completion and read-out subgroups showed similar internal consistency and item selectivity scores. With regard to convergent validity, significant correlations were found between Wound-QoL and EQ-5D-3L (T0: r = − 0.45, *p* < 0.001, T1: r = − 0.50, *p* < 0.001) as well as between Wound-QoL and pain VAS (T0: r = 0.23, *p* = 0.012, T1: r = 0.37, *p* = 0.001) at both time points. Responsiveness analyses showed significant correlations between changes in Wound-QoL and changes in EQ-5D-3L (r = − 0.37, *p* < 0.001), pain VAS (r = 0.24, *p* = 0.044) and wound size (r = 0.24, *p* = 0.013). The self-completion and read-out subgroups showed differences in convergent validity and responsiveness.

**Conclusions:**

The results indicate that the Dutch version of the Wound-QoL has positive psychometric properties. However, more research is needed to further explore the differences between self-completed and read-out questionnaires.

## Background

Chronic wounds are lesions of the skin and subcutaneous tissue that show insufficient healing two to four weeks after occurrence according to the Dutch recommendation [1]. They may have various causes, such as venous or arterial insufficiency, diabetes mellitus, trauma, malignancy, self-mutilation or physical pressure [1]. A meta-analysis [2] including several worldwide studies showed a pooled prevalence of 2.21 per 1000 persons for chronic wounds of mixed aetiologies. A slightly higher annual prevalence of approximately 500,000 patients was found in the Netherlands [3]. However, one must be careful not to compare prevalence rates with each other, because there is no uniform definition for the term ‘chronic wound’. The prevalence of chronic wounds increases with age. The highest prevalence rates occur in people above the age of 65 [4]. Therefore, prevalence rates are likely to increase due to the ageing of the Dutch population [5].

Patients with chronic wounds often experience impairments such as pain, the necessity for frequent dressing changes and mobility limitation, which negatively impact the patients’ health-related quality of life (HRQoL) [6, 7].

HRQoL is a multidimensional (e.g. physical, psychological, social) construct defined as the health-related functioning and well-being of a person [8]. Due to its subjectivity, HRQoL is assessed as patient-reported outcome (PRO). It is considered an important outcome measure of medical treatment in addition to the clinical outcome measures [9]. Nowadays, HRQoL questionnaires are widely used and play a major role in healthcare decisions and treatment evaluation [10]. The use of validated questionnaires to measure patient characteristics and treatment outcomes from the patients’ perspective is necessary in the treatment of chronic wounds as well [11–13]. However, a validated, short, and easy-to-use HRQoL questionnaire for clinical use is not available in the Netherlands.

In view of the age composition of the patient group with chronic wounds as well as the necessity to regularly assess HRQoL in routine care, brief and easy-to-use questionnaires are recommended [14].

For this purpose, the Wound-Qol questionnaire [12] has been developed based on three more extensive questionnaires for patients with wounds (the Cardiff Wound Impact Schedule (CWIS) [13], the Freiburg Life Quality Assessment for wounds (FLQA-W) [11] and the Wuerzburg Wound Score (WWS) [15]). The Wound-QoL [12, 15, 16] consists of 17 questions from which a total scale score and three subscale scores can be calculated with higher values indicating worse HRQoL. The subscale ‘body’ is derived from items 1 to 5 (e.g., “my wound hurt”, “the wound has affected my sleep”), the subscale ‘psyche” is derived from items 6 to 10 (e.g., “the wound has made me unhappy”, “I have been afraid of knocking the wound”), and subscale ‘everyday life’ is derived from items 11 to 16 (e.g., “I have had trouble moving around because of the wound”, “the wound has limited my leisure activities”). Item 17 does not belong to neither of the subscales. The Wound-QoL was developed to create a brief instrument. The Wound-QoL questionnaire has been translated into 20 languages (available on the website: www.wound-qol.com). However, a validated Dutch translation of the Wound-QoL has not yet become available.

Therefore, the aim of this study was to translate and validate the Wound-QoL questionnaire for Dutch people suffering from chronic wounds.

## Methods

We translated the validated German Wound-QoL questionnaire into Dutch according to the international standards for cross-cultural adaptations of outcome instruments [17]. The translation process included two forward and two backward translations and harmonization of these versions.

In the pre-test, it became obvious that the Dutch version of the item “the wound has affected my sleep” did not suit the response scale. Therefore, slight alterations have been made so that this item keeps the same meaning but suits the response options better (from “kon ik door de wond niet goed slapen” to “had ik door de wond problemen met slapen"). Accordingly, the present study validated the Wound-QoL including this minor change.

In order to validate the Dutch version of the Wound-QoL, we recruited patients with chronic wounds (of different aetiologies) who were able to speak and understand Dutch and were 18 years or older. Patients had to have a wound at both study inclusion and after six weeks. Only one exclusion criterion was defined (i.e., having a healed wound according the Dutch supported definition1 [1] of chronic wounds within the six weeks of study participation) to achieve a relatively heterogenous sample reflecting the target group of the Wound-QoL. Patients were recruited from a home care organization providing wound care for general practitioners and different wound centres in the Netherlands, which are part of both academic and non-academic hospitals. The aim was to recruit no fewer than 100 patients, which is considered an adequate sample size for analyzing various psychometric properties [18]. Recruitment took place from August 2018 to May 2019.

Patients were asked to complete the questionnaires during their visits at the moment of inclusion (T0) and after six weeks (T1). The first questionnaire consisted of sociodemographic questions (weight, height, number of people in the household), the Dutch version of the Wound-QoL, the generic HRQoL instrument EQ-5D-3L and a visual analogue scale (VAS) measuring the patients’ worst pain during the day. The EQ-5D-3L is a short questionnaire assessing generic HRQoL using five questions about mobility, self-care, usual activities, pain, and anxiety/depression. Each question is answered on a three level scale representing no problems, some problems, and extreme problems. Additionally, the questionnaire encompasses a VAS about the patient’s self-rated health [19]. The second questionnaire consisted of the same instruments except sociodemographic questions. Further data were obtained from medical records (age, gender, smoking habits, compression therapy, wound diagnosis, medication, comorbidities). Additionally, the size of the wound surface was measured at T0 and T1 by using a camera (InSight®). For patients with various wounds, the size of the largest wound was measured.

The majority of patients completed the questionnaires by themselves (self-completion group). Other patients were also willing to answer the questionnaires but were not able or did not wish to complete the questionnaire by themselves. Reasons were, for example, that patients did not have their glasses with them or that it would burden them to read the questionnaires on their own. In these cases, nurses read out the questionnaires to the patients and ticked the according response options (read-out group).

For a small subgroup of participants, the time of completion was measured.

All patients gave prior written informed consent to participate in the study. The study has been approved by the medical ethical committee of the Isala Clinics (No. 180916; Zwolle, The Netherlands).

Statistical analyses were performed with SPSS Statistics version 25 (IBM®, Armonk, NY). The Wound-QoL scores were calculated by averaging the respective items, if at least 75% of the items had been answered. The EQ-5D-3L index was calculated by using the utility algorithm for the Netherlands. The following properties were analyzed in order to validate the Dutch Wound-QoL questionnaire: floor and ceiling effects (i.e. percentage of patients with the highest/lowest scores), internal consistency (i.e. Cronbach’s alpha for the global scale and for each subscale), item selectivity (i.e. correlation of the global score with each item; correlation of the subscale scores with each respective item; correlation of the global score with the subscale scores), convergent validity (i.e. correlation of the global score with the EQ-5D-3L score, score of pain VAS, the size of the wound surface), and responsiveness (i.e. correlation of changes between T0 to T1 in the global score with changes in the EQ-5D score, VAS score and the size of the wound surface). For convergent validity and responsiveness, a generic HRQoL has been chosen as other wound-specific questionnaires were not available in Dutch at the time of study conduct. To account for the specific burden posed by chronic wounds, wound pain and wound size have additionally been included as convergent criteria. Normal distribution of items and scores was tested using the one-sample Kolmogorov-Smirnov test. As Wound-QoL scores and items showed no normal distribution according to this test, non-parametric Spearman correlation was calculated. According to Cohen [20], a correlation coefficient will be interpreted as small when r = 0.1, as moderate when r = 0.3, and as large when r = 0.5. For internal consistency, Cronbach’s alpha of 0.7 can be considered acceptable and 0.9 can be considered good [21]. In order to account for possible bias caused by the different modes of completion (self-completion; read-out), we compared the sociodemographic aspects of both subgroups using unpaired t-test and Chi-squared tests, and we conducted each analysis in both the total sample and each subgroup.

For convergent validity, hypotheses were formulated about the direction and relative strength magnitude of the correlations between the Wound-QoL score comparator instruments. It was hypothesized that higher EQ-5D-3L scores would be associated with lower Wound-QoL scores, whereas higher pain VAS scores and larger wound size would be associated with higher Wound-QoL scores. With regard to the relative magnitude strength of the correlations, it was assumed that the correlation between Wound-QoL scores and EQ-5D-3L scores were highest, because both instruments represent multidimensional HRQoL constructs. It was assumed that the correlation between Wound-QoL and pain VAS was the second strongest as pain is a major cause of limitation for patients [22]. The weakest correlation was expected to be found between Wound-QoL and the wound size, as wound size itself does not cause major restrictions and burden compared to other aspects of the wound, such as pain or visibility. With regard to responsiveness, it was hypothesized that the directions and relative magnitudes of change in the instruments would correspond to those of convergent validity.

## Results

We included 120 patients. Sixty-four of them (53.3%) completed the questionnaire by themselves. To 55 of them (45.8%), nurses read out the questionnaire. For one patient (0.8%), there is no information about the mode of questionnaire completion. The gender distribution was almost equal (*n* = 63, 52.5% women). The patients had an average age of 73 ± 14 years (min = 18, max = 98). On average, the wound size decreased from 9.88 cm^2^ at T0 to 6.84 cm^2^ at T1 (Table 1). The most common diagnoses (Table 2) were diabetic foot ulcer (*n* = 37, 30.8%), venous ulcer (*n* = 20, 16.7%) and ulcer caused by trauma (*n* = 19, 15.8%). The majority of wounds were located on the lower legs (*n* = 61, 50.8%) and feet (*n* = 48, 40.0%). The most frequent comorbidities (Table 2) were cardiovascular diseases (*n* = 81, 67.5%), diabetes (*n* = 58, 48.3%) and peripheral vascular diseases (*n* = 53, 44.2%).

**Table 1:** Sociodemographic and wound-specific descriptive statistics for the total sample and subsamples

	Total sample (***N*** = 120)		Self-completion (***N*** = 64)		Read-out (***N*** = 55)		Self-completion vs. read-out
	n	%	n	%	n	%	***p***-value^**1**^
Male (vs. female)	57	47.5	34	53.1	22	40.0	0.153
Smoker (yes)	21	17.5	10	15.6	10	18.5	0.676
Living alone (yes)	48	40.0	21	32.8	27	49.1	0.071
Compression therapy (yes)	69	57.5	34	53.1	34	61.8	0.339
	**M**	**SD**	**M**	**SD**	**M**	**SD**	**p-value**^**2**^
Age [years]	73.23	14.24	68.89	14.71	78.42	11.99	< 0.001
Length [m]	1.72	0.10	1.73	0.10	1.70	0.11	0.183
Weight [kg]	82.52	19.85	84.36	17.03	80.00	22.79	0.238
BMI [kg/m^2^]	27.75	5.48	28.11	4.74	27.30	6.33	0.427
Wound duration [weeks]	34.55	65.02	22.28	39.61	49.50	84.43	0.023
Wound size at T0 [cm^2^]	9.88	21.33	9.39	13.69	10.57	27.89	0.766
Wound size at T1 [cm^2^]	6.84	18.67	7.33	15.05	6.36	22.42	0.786
Number of medications	8.60	4.39	8.56	4.72	8.49	3.89	0.929
Number of comorbidities	2.87	1.37	2.83	1.42	2.87	1.31	0.860

^1^ p-value according to Chi-squared tests, ^2^ p-value according to unpaired t-test

**Table 2:** Wound diagnoses and comorbidities at baseline

	Frequency	Percentage
Wound diagnoses		
Diabetic foot ulcer	37	30.8
Venous ulcer	20	16.7
Traumatic ulcer	19	15.8
Arterial ulcer	14	11.7
Malignant ulcer	8	6.7
Mixed ulcer	6	5.0
Pressure ulcer	5	4.2
Vasculitis	1	0.8
Other	10	8.3
Comorbidities^a^		
Cardiovascular diseases	81	67.5
Diabetes	58	48.3
Peripheral vascualar diseases	53	44.2
Chronic obstructive pulmonary disease	26	21.7
Rheumatism	25	20.8
Renal failure	21	17.5
Oncologic disease	17	14.2
Thrombosis/pulmonary embolism	14	11.7
Surgical intervention	13	10.8
Neurological disorders	11	9.2
Thyroid disorders	10	8.3
Psychiatric disorders	8	6.7
Other	12	10.0

^a^Percentages do not sum up to 100.0% because multiple answers were possible

Descriptive statistics of the Wound-QoL items, the global scale, and the subscale can be seen in Table 3.

**Table 3:** Descriptive statistics of Wound-QoL items, global scale, and subscales at the first and second visit

Wound-QoL items and scales		T0					T1				
	In the last seven days …	n	min	max	mean	SD	n	min	max	mean	SD
1	my wound hurt	120	0.00	4.00	1.51	1.230	119	0.00	4.00	1.35	1.266
2	my wound had a bad smell	119	0.00	4.00	0.34	0.807	120	0.00	4.00	0.29	0.782
3	the discharge from the wound has upset me	119	0.00	4.00	0.90	1.182	120	0.00	4.00	0.73	1.075
4	the wound has affected my sleep	120	0.00	4.00	1.18	1.288	118	0.00	4.00	0.86	1.233
5	the treatment of the wound has been a burden to me	118	0.00	4.00	0.92	1.207	118	0.00	4.00	0.82	1.159
6	the wound has made me unhappy	118	0.00	4.00	0.93	1.238	120	0.00	4.00	0.80	1.149
7	I have felt frustrated because the wound is taking so long to heal	120	0.00	4.00	1.58	1.406	120	0.00	4.00	1.72	1.379
8	I have worried about my wound	119	0.00	4.00	1.42	1.453	118	0.00	4.00	1.27	1.400
9	I have been afraid of the wound getting worse or of getting new wounds	120	0.00	4.00	1.36	1.431	120	0.00	4.00	1.21	1.289
10	I have been afraid of hitting the wound against something	119	0.00	4.00	1.61	1.445	119	0.00	4.00	1.34	1.337
11	I have had trouble moving around because of the wound	117	0.00	4.00	1.50	1.362	117	0.00	4.00	1.23	1.373
12	climbing stairs has been difficult because of the wound	85	0.00	4.00	1.47	1.385	90	0.00	4.00	1.13	1.400
13	I have had trouble with everyday activities because of the wound	120	0.00	4.00	1.55	1.466	120	0.00	4.00	1.23	1.288
14	the wound has limited my recreational activities	120	0.00	4.00	1.74	1.520	120	0.00	4.00	1.49	1.402
15	the wound has forced me to limit my contact with other people	120	0.00	4.00	1.64	1.549	118	0.00	4.00	1.47	1.424
16	I have felt dependent on help from others because of the wound	120	0.00	4.00	1.80	1.447	120	0.00	4.00	1.52	1.372
17	the wound has been a financial burden to me	120	0.00	4.00	0.62	1.161	119	0.00	4.00	0.48	0.910
Global score		120	0.06	3.35	1.29	0.788	120	0.00	3.24	1.12	0.785
Subscale ‘body’		120	0.00	3.60	0.98	0.765	120	0.00	3.00	0.81	0.748
Subscale ‘psyche’		120	0.00	3.80	1.38	1.031	120	0.00	4.00	1.27	0.987
Subscale ‘everyday life’		119	0.00	4.00	1.62	1.213	117	0.00	4.00	1.36	1.131

*T0* first visit, *T1* second visit, *N* number of patients completing the item/for whom the respective scale could be calculated, *min* minimum, *max* maximum, *SD* standard deviation

### Number of missing values

Of the 17 items, nine items at T0 and eight items at T1 showed no missing values. One item (‘climbing stairs has been difficult because of the wound’) showed a large number of missing values (T0: 29.2%, T1: 25.0%). All patients with missing values for this item filled in or stated during the interview that this item was not applicable to their situation, but this was not a response option in the Wound-QoL. For the remaining items, the number of missing values ranged from 0.8 to 2.5% at both time points equalling to one to three patients per item. Regarding the global and subscale scores, only for one patient at T0 and three patients at T1 the subscale ‘everyday life’ could not be calculated because of too many missing values.

### Floor and ceiling effects

The global score showed no ceiling effect at either time point and a minor floor effect only at T1 (0.8%). Although the ‘body’ and ‘psyche’ subscales did not show ceiling effects at T0, the ‘psyche’ subscale showed a minor ceiling effect at T1 (0.8%). The ‘everyday life’ subscale showed minor ceiling effects at both time points (T0: 1.7%, T1: 3.4%). All subscales showed floor effects at both T0 (body: 12.5%, psyche: 9.2%, everyday life: 6.7%) and T1 (21.7, 7.5, 11.1% respectively). Floor effects in ‘body’ and ‘everyday life’ subscales were less pronounced in the patients who completed the questionnaire by themselves (T0: 6.3, 4.7%, T1: 18.8, 8.1% respectively) compared to the patients in the read-out group (T0: 20.0, 9.3%, T1: 25.5, 14.8% respectively).

### Changes in the mean scores

The mean values decreased over time for both Wound-QoL global score (T0: 1.29, T1: 1.12) and Wound-QoL subscale scores (body: T0: 0.98, T1: 0.81, psyche: T0: 1.38, T1: 1.27, everyday life: T0: 1.59, T1: 1.36). The t-test results revealed significant improvements for the global score (t (119) = 2.566, *p* = 0.012), the ‘body’ subscale (t (119) = 2.221, *p* = 0.028) and the ‘everyday life’ subscale (t (119) = 2.500, *p* = 0.014), but not for the ‘psyche’ subscale (t (119) = 1.136, *p* = 0.258).

When we consider each of the subgroups, a significant change was observed in the self-completion group (global score: *p* = 0.005, body: *p* = 0.038, everyday life: *p* = 0.002, psyche: *p* = 0.076), but not in the read-out group (global score: *p* = 0.820, body: *p* = 0.371, everyday life: *p* = 0.783, psyche: *p* = 0.404).

### Internal consistency

The internal consistency of the Wound-QoL global score was high at both times points (T0: α = 0.889, T1: α = 0.918). With regard to the subscales, the internal consistency was highest for the ‘everyday life’ subscale (T0: α = 0.895, T1: α = 0.925), followed by the ‘psyche’ subscale (T0: α = 0.794, T1: α = 0.811) and the ‘body’ subscale (T0: α = 0.673, T1: α = 0.687). The self-completed and read-out questionnaires showed the same patterns.

### Item selectivity

The item selectivity of the items of the global score ranged from r = 0.251 to r = 0.768 at T0 and from r = 0.395 to r = 0.793 at T1. The items with the highest correlation coefficients were: ‘I have had trouble with everyday activities because of the wound’ (T0: r = 0.768, T1: r = 0.793), ‘the wound has limited my recreational activities’ (T0: r = 0.760, T1: r = 0.723), ‘the wound has forced me to limit my contact with other people’ (T0: r = 0.754, T1: r = 0.727) and ‘I have had trouble moving around because of the wound’ (T0: r = 0.712, T1: r = 0.728). It should also be noted that these four items showed the highest correlation coefficients in both the self-completion and the read-out group.

The item selectivity for the ‘body’ subscale ranged from r = 0.369 to r = 0.769 at T0 and from r = 0.515 to r = 0.775 at T1; for the ‘psyche’ subscale, it ranged from r = 0.677 to r = 0.778 at T0 and from r = 0.593 to r = 0.807 at T1 and for the ‘everyday life’ subscale, it ranged from r = 0.703 to r = 0.890 at T0 and from r = 0.707 to r = 0.870 at T1. The correlation between the global scale and subscales was highest for the ‘everyday life’ subscale (T0: r = 0.867, T1: r = 0.874), followed by the ‘psyche’ subscale (T0: r = 0.801, T1: r = 0.801) and the ‘body’ subscale (T0: r = 0.632, T1: r = 0.689).

Item selectivity generally showed minor effects and was similar for both the self-completion and the read-out subgroup.

### Convergent validity

The correlation between EQ-5D-3L and Wound-QoL was significant (T0: r = − 0.451, *p* < 0.001, T1: r = − 0.501, *p* < 0.001). The same accounts for the correlation between pain VAS and Wound-QoL (T0: r = 0.232, *p* = 0.012, T1: r = 0.372, *p* = 0.001). Although the correlation between the wound size and Wound-QoL was significant at T1 (r = 0.228, *p* = 0.015), it was not significant at T0 (r = 0.124, *p* = 0.178). These correlations with the EQ-5D-3L represent moderate to large effect sizes, whereas the other correlations represent small to moderate effect sizes [20].

For the self-completion subgroup, the correlation between EQ-5D-3L and Wound-QoL was significant at both time points (T0: r = − 0.611, *p* < 0.001, T1: r = − 0.501, *p* < 0.001). For the read-out subgroup, the correlation between EQ-5D-3L and Wound-QoL was significant at both time points as well (T0: r = − 0.306, *p* = 0.023, T1: r = − 0.556, *p* < 0.001). Additionally, for the read-out subgroup, the correlation between pain VAS and Wound-QoL was significant at both time points (T0: r = 0.357, *p* = 0.008, T1: r = 0.486, *p* = 0.003). The correlations with the EQ-5D-3L again represent moderate to large effect sizes, whereas the correlations with the pain VAS represent moderate effect sizes [20]. Table 4 shows the results regarding convergent validity for the total group and the subgroups.

**Table 4:** Convergent validity of the Wound-QoL global score

	EQ-5D-3L		Pain VAS		Wound size	
	T0	T1	T0	T1	T0	T1
Total sample						
r	**−0.451**	**−0.501**	**0.232**	**0.372**	0.124	**0.228**
p-value	**< 0.001**	**< 0.001**	**0.012**	**0.001**	0.178	**0.015**
n	**119**	**119**	**116**	**75**	119	**113**
Subgroup: Self-completion						
r	**−0.611**	**− 0.501**	0.180	0.306	0.143	0.242
p-value	**< 0.001**	**< 0.001**	0.165	0.055	0.263	0.063
n	**63**	**64**	61	40	63	60
Subgroup: Read-out						
r	**−0.306**	**−0.556**	**0.357**	**0.486**	0.032	0.212
p-value	**0.023**	**< 0.001**	**0.008**	**0.003**	0.816	0.132
n	**55**	**54**	**54**	**35**	55	52

Significant results are marked bold; *r* Spearman correlation coefficient, *n* number of patients, *VAS* visual analogue scale

### Responsiveness

Significant correlations were found between changes in Wound-QoL and changes in EQ-5D-3L (r = − 0.373, *p* < 0.001), changes in pain VAS (r = 0.239, *p* = 0.044) and changes in wound size (r = 0.235, *p* = 0.013). Although the effect sizes were moderate for correlations between changes in Wound-QoL and changes in EQ-5D-3L, the effect sizes were small for the correlations between changes in Wound-QoL and changes in pain VAS and wound size.

For the self-completion subgroup, only the correlation between changes in Wound-QoL and changes in EQ-5D-3L was significant (r = − 0.408, *p* = 0.001). The effect size was moderate. For the read-out subgroup, the correlation between changes in Wound-QoL and changes in EQ-5D-3L (r = − 0.285, *p* = 0.037), as well as the correlation between changes in Wound-QoL and changes in wound size (r = 0.290, r = 0.037), were significant, each representing small effect sizes. Table 5 shows the results regarding responsiveness for the total sample and the subgroups.

**Table 5:** Responsiveness of the Wound-QoL global score

	EQ-5D-3L	Pain VAS	Wound size
Total sample			
r	**−0.373**	**0.239**	**0.235**
p-value	**< 0.001**	**0.044**	**0.013**
N	**118**	**71**	**112**
Subgroup: self-completing			
r	**−0.408**	0.293	0.184
p-value	**0.001**	0.078	0.162
N	**63**	37	59
Subgroup: read-out			
r	**−0.285**	0.154	**0.290**
p-value	**0.037**	0.383	**0.037**
n	**54**	34	**52**

Significant results are marked bold; *r* Spearman correlation coefficient, *n* number of patients, *VAS* visual analogue scale

### Time of completion

For nine patients, the time needed to complete the Wound-QoL questionnaire was recorded. The time needed ranged from 0:57 min (self-completion) to 3:53 min (read out) at T0.

## Discussion

The aim of this study was to translate the Wound-QoL questionnaire into Dutch and to test the validity of the translated version. Overall, the results showed that the Dutch version of the Wound-QoL is a valid instrument that only takes little time to complete. It showed a good internal consistency and a small to moderate yet significant convergent validity with the EQ-5D-3L for the total sample. These results are similar to those from the validation study of the Swedish Wound-QoL [23]. Additionally, the results of the present study are similar with the results regarding the German original version [24].

Similar to previous studies [23], only the item about 'climbing stairs' showed a high number of missing values. Patients who completed the questionnaire by themselves often added a comment next to this item. Patients to whom the questionnaire was read out expressed during the interview with a nurse that this item did not apply to them (e.g. because climbing the stairs was not part of their daily routine). However, further analyses (not shown in the results section) revealed that the exclusion of this item would not impact the overall results.

For convergent validity and responsiveness analyses, other Dutch wound-specific questionnaires were not available at the time of the study conduct. Therefore, a generic HRQoL instrument (EQ-5D-3L) and wound-specific clinical data were used. Overall, formulated hypotheses were confirmed. Significant yet moderate correlations between Wound-QoL and the pain VAS show that wound pain is not the only wound characteristic influencing disease-specific HRQoL. Here it needs to be considered that pain might not only be caused by the wound itself but also by wound-related factors, such as wound dressing [25]. The Wound-QoL does not differentiate between different sources of pain. If patients report high impairment in the pain item (or in fact, in any item), it is important to discuss the nature of impairments with the individual patient in order to optimize wound care [26]. Stronger correlations between generic and wound-specific HRQoL show that both types of HRQoL adequately reflect an overall picture of the patient’s situation. However, differences between these constructs underlined that the generic HRQoL is influenced by other aspects than the wound as well. Additionally, convergent validity analyses showed no significant correlation between Wound-QoL and wound size. This could mean that the physical impact (e.g. pain, odour) and the visible impact (e.g. exudate) of a wound is more burdensome than the wound size itself [15]. However, improvements in any of these characteristics (generic HRQoL, pain, wound size) were correlated with improvements in the wound-specific HRQoL according to the Wound-QoL.

For several psychometric properties, we observed differences between the patients who completed the questionnaire by themselves and those to whom the questionnaire was read out. With regard to floor effects, change in mean scores, convergent validity and responsiveness in particular, discrepancies were found between the two subgroups. The sample characteristics showed that the patients in the read-out subgroup were significantly older and had wounds of longer duration than the other subgroup. Especially, the longer wound duration in the read-out group might explain the absence of significant HRQoL change in this group, because the longer a wound persists the more likely is that it is a particularly hard-to-heal wound that is less likely to show improvement. An alternative explanation may be that patients with shorter wound duration are still adapting to the situation, which can improve their HRQoL [27]. Overall, it cannot be decided whether the mode of completion changed the validity of the questionnaire or whether structural differences between the two subgroups were causing these discrepancies. This could be investigated in future studies, in which a patient sample is randomized into a self-completion and a read-out group. In order to minimize this potential bias when using the Wound-QoL, read-out completion should be as similar to self-completion as possible by closely sticking to the questionnaire text. If possible, individual patients should use the same mode every time they complete the questionnaire in order to ensure comparability over time.

Finally, it should be noted that this validation study analyzed psychometric properties of the total scale and subscales, which serves research and evaluation purposes in particular. However, we recommend that the patients’ responses are considered on an item level for routine care purposes as well as each aspect can be of great importance for individuals [7]. Therefore, the patients’ responses to items about physical burden (e.g. odour, exudate), emotional burden (e.g. frustration, worries) and limitations in activities of daily living (e.g. leisure activities, contact with others) should be considered for shared decision-making and joint goal setting.

One of the strengths of this study is that we reached the targeted size of the total sample and each subgroup (self-completion and read-out) consisted of more than 50 patients, representing a good sample size for analysing psychometric properties [18]. At the time of study conduction, no other wound-specific HRQoL questionnaire had been translated into Dutch. In the meanwhile, the CWIS had been translated into and validated in Dutch [28]. However, this is a comparably long questionnaires and the authors of the Dutch CWIS study mention the necessity of a short instrument. Accordingly, this was the first translation and validation of a short and easy-to-use Dutch HRQoL questionnaire, which is promising for future clinical care and research.

A limitation of this study is the phrasing of one item (“the wound has affected my sleep”) being slightly changed in order to better fit the response options. Therefore, the present Dutch version of the Wound-QoL slightly differed from the standardized translation process [29]. Another limitation was that time of completion was measured for only a small subgroup of patients. As some patients expressed to feel under time pressure when we took their time of completion, we decided to stop time measurement because this was not a primary research question. The most frequent wound aetiology were diabetic foot ulcers, despite venous leg ulcers being the most prevalent aetiology in Western countries. This was caused by the fact that enrolled patients were treated by a vascular surgeon, whereas patients with venous leg ulcers are more often treated by dermatologists in the Netherlands. As HRQoL and pain might differ with regard to the underlying aetiology, this might lead to some deviations from patients in other settings [25].

Overall, this study indicates that the Dutch Wound-QoL questionnaire is a valid instrument for measuring the HRQoL of patients with chronic wounds. However, the study also shows different outcomes between self-completed and read-out questionnaires. In further studies, the validity of different modes of questionnaire completion should be investigated. Furthermore, this should raise awareness about new modes of questionnaire completion for people who are not able to complete questionnaires themselves.

## Conclusions

The results indicate that the Dutch version of the Wound-QoL has positive psychometric properties. However, more research is needed to further explore the differences between self-completed and read-out questionnaires.

## Data Availability

On request from corresponding author.

## Abbreviations

CWIS: Cardiff Wound Impact Schedule
FLQA-W: Freiburg Life Quality Assessment for wounds
HRQoL: Health-related quality of life
n: number of patients
PRO: Patient-reported outcome
p: significance level
r: Spearman correlation coefficient
T0: Patients were asked to complete the questionnaires during their visits at the moment of inclusion
T1: Patients were asked to complete the questionnaires during their visits after six weeks
VAS: Visual analogue scale
WWS: The Wuerzburg Wound Score
